# Treatment Sequencing Strategies in Lung Cancer

**DOI:** 10.3779/j.issn.1009-3419.2022.104.01

**Published:** 2022-05-20

**Authors:** Daniel Humberto Pozza, Ramon Bezerra Andrade de Mello

**Affiliations:** 1 Department of Biomedicine, Faculty of Medicine and i3s, University of Porto, 4200-319 Porto, Portugal; 2 Discipline of Medical Oncology, Post-graduation Program in Medicine, Nine of July University (UNINOVE), São Paulo, Brazil./Nine of July Hospital, São Paulo, Brazil

**Keywords:** Lung neoplasms, Immunotherapy, Clinical trials, Targeted therapies, Pembrolizumab, Nivolumab, Atezolizumab, Necitumumab, Brigatinib

## Abstract

**Background and objective:**

The advances in the lung cancer screening methods and therapeutics, together with awareness towards deleterious habits, such as smoking, is increasing the overall survival with better quality of life for the patients. However, lung cancer is still one of the most common and fatal neoplasm with a high incidence and consequently burden to public health worldwide. Thus, based on guidelines and recent phases Ⅱ and Ⅲ clinical trials studies, this manuscript summarizes the current treatment sequencing strategies in lung cancer.

**Methods:**

A comprehensive search of related articles was performed focused on phases Ⅱ and Ⅲ clinical trials studies.

**Results:**

The lung cancer management should take into consideration the tumor characteristics, histology, molecular pathology and be discussed in a multidisciplinary team. Lung cancer treatment options comprises surgery whenever possible, radiotherapy associate with/or chemotherapy and immunotherapy as monotherapy, or combined with chemotherapy and best palliative care.

**Conclusions:**

The screening predictability in more patients, smoking reduction, early diagnosis, better disease understanding and individualized, more effective and tolerable therapeutics are related to an increasing in overall survival and quality of life. In the near future improvement of personalized therapy in precision medicine is expected, enhancing new predictive biomarkers, optimal doses and optimal treatment sequencing as well as anti-cancer vaccines development.

## Introduction

Lung cancer is one of the most common neoplasm with a high mortality rate, representing a global burden to public health worldwide leading to disabilities and premature mortality since few patients will survive longer than 5 years. The malignant behavior and lack of cure leads to physical impairment and psychological distress with marked reduced quality of life, requiring a multidisciplinary and complex treatment^[1-7]^.

The smoking reduction is responsible for the falling incidence of lung cancer, particularly in men. The early diagnosis, better disease understanding and more effective and tolerable therapeutics are related to an increasing in survival. The screening predictability in more patients, being diagnosed with earlier stages of the disease, are also increasing the candidates for surgery. The advances in histopathology, biomarkers and new genetics tools are helping to choose the most appropriate therapy^[6, 8-12]^. The most predictive biomarkers are anaplasic lymphoma kinase (*ALK*) fusion oncogene, *ROS1* gene rearrangements, mutant epidermal growth factor receptor (*EGFR*) kinases, human epidermal growth factor receptor-2 (*HER2*) and *BRAF* mutations, *RET* gene rearrangements, and high-level *MET* amplifications. Therapeutic advances, such as biomarker testing results should be expedited in order to prevent treatment delays, improving survival^[8, 13]^.

The recommended initial lung cancer workup should include computed tomography and magnetic resonance imaging and pathologic tests, to determine the tumor subtype with biomarkers, such as programmed death-ligand 1 (PD-L1) immunohistochemistry. *EGFR*, *ALK*, *ROS1*, *BRAF*, *RET*, *METs* or *HER2* are also recommended in patients with non-squamous histology whenever possible and when next-generation sequencing is used^[8]^.

Lung cancer approach and treatment should be based on patient status that includes medical history with comorbidities, physical examination, lungs capacity, cardiac risk, age, weight loss, performance status (PS) and preferences. The management should take into consideration the tumor characteristics, histology, molecular pathology and be discussed together with a multidisciplinary team ^[14-17]^. Lung cancer is potentially curable when limited in stage by surgery. However, this is not possible for most cases and radiotherapy associate with/or chemotherapy are usually employed. For patients without an actionable driver mutation and when targeted therapies are not available, chemotherapy was the standard of care. Nowadays immunotherapy, mainly programmed death-1 (PD-1)/PD-L1 blockade immunotherapy, as monotherapy, or combined with chemotherapy is the standard of care because of survival benefits and less adverse events such as fatigue, nausea, diarrhea, decreased appetite and asthenia. Furthermore, anemia, alopecia, neutropenia, myalgia, and stomatitis are adverse events attributed to chemotherapy only. On the other side, immunotherapy toxicity is more associated with hypothyroidism, hyperthyroidism, pneumonitis and rash, although they rarely occur ^[1, 18-22]^.

Based on guidelines and recent phases Ⅱ and Ⅲ clinical trials studies, the objective of this review was to describe the current treatments of initial and advanced lung cancer through surgery, chemotherapy, immunotherapy, radiotherapy, and/or targeted therapy.

## Methods

A comprehensive search of related articles was performed in PubMed.gov using Mesh Terms: "Lung Neoplasms"[Mesh] AND "Clinical Trial, Phase Ⅱ" [Publication Type] AND "Clinical Trial, Phase Ⅲ" [Publication Type] as well as ("Lung Neoplasms"[Mesh]) AND "Guideline" [Publication Type]. Additionally, some filters were selected including "Humans" in Species, "English" in Language and "Clinical trial" or "Review" in Article Type according to the Mesh Terms used. The manuscripts search was performed between April and June of 2021. The two readers carefully screened all articles obtained from the reported search initially based on titles and abstracts. Whenever no sufficient information in the title/abstract to allow decision making regarding inclusion or exclusion criteria, the article was evaluated only after full text was obtained and reviewed in order to make a final decision. Any disagreement between the two investigators were solved by consensus. Screening the reference lists of the selected articles complemented the search with additional manuscripts to be evaluated. The inclusion criteria comprised mainly up-to-date human clinical trials or reviews focused in guidelines based on human clinical trials. For the eligibility of the study, the full texts were accessed by extracting the data regarding the methods, participants, intervention and outcomes by both investigators, independently for discussion. The exclusion criteria included *in vitro* studies, outdated protocols, no full text in English or duplicated studies.

## Results

**Figure 1:**
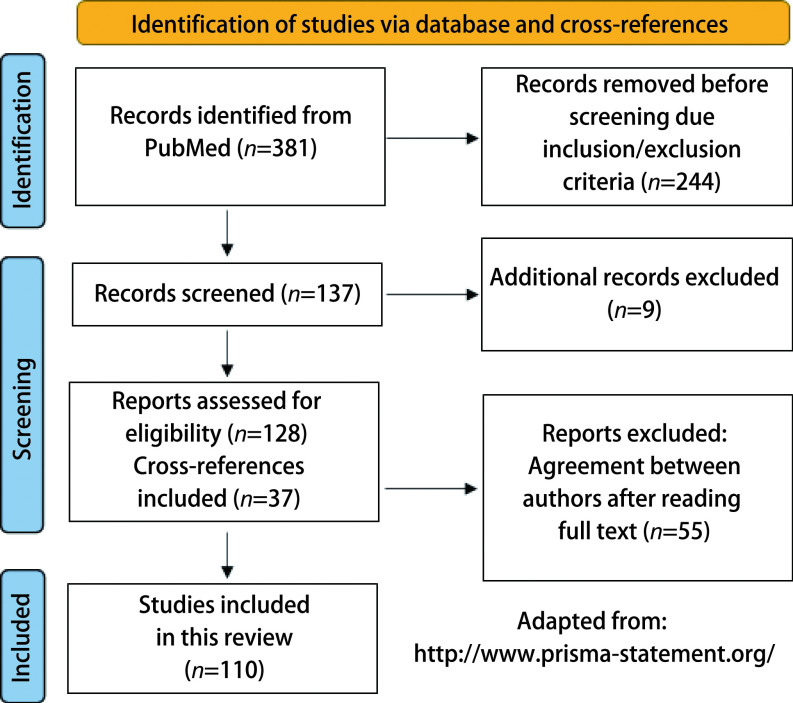
Flow diagram of manuscript search adapted from PRISMA.

In the first search, 381 articles were obtained and 244 articles were excluded after inclusion/exclusion criteria were employed. In the process of full texts assessments 9 manuscripts were also excluded by the two authors after reading abstracts and/or main texts. A total of 128 manuscripts were fully evaluated and 55 were exclude after reading and discussing the contents. In addition, after screening the reference lists of these 128 selected articles, 37 other manuscripts that did not appear in the first search, were also included. The two authors of the present review carefully evaluated, as many times as necessary, the 174 selected articles finally excluding 64 of them. Therefore, a total of 110 manuscripts were used in the present review. The flow diagram (Fig 1) describes the results of the manuscript search. Statistical analysis was performed with SPSS 27.0 and confirmed the high agreement between researches (Kappa=0.88).

PRISMA: Preferred Reporting Items for Systematic Reviews and *Meta*-Analyses, n: number of studies.

Among the 110 included articles, 38 phases Ⅱ or Ⅲ clinical trials were selected, being 6 related to the small cell lung cancer (SCLC) treatment (Tab 1) and 24 to the non-small cell lung cancer (NSCLC) treatment (Tab 2). Additionally, 18 phases Ⅱ or Ⅲ clinical trials with focus on advanced NSCLC and molecular profile for gene mutations were also evaluated (Tab 3). These phases Ⅱ or Ⅲ clinical trials were organized in separate tables in comprehensive analysis section to facilitate comparisons.

**Table 1:** Phases Ⅱ or Ⅲ clinical trials related to the SCLC treatment

Reference	Brief study methods	Relevant key findings
Horn *et al* (2018)	Phase Ⅲ multinational trial: carboplatin and etoposide with either atezolizumab or placebo in SCLC without previously treatment	Atezolizumab+chemotherapy=significantly longer overall survival and progression-free survival
Goto *et al* (2016)	Phase Ⅲ trial: chemotherapy+cisplatin, etoposide, and irinotecan VS topotecan monotherapy as second-line chemotherapy in patients with sensitive relapsed SCLC in Japan	The proposed combination can be considered the standard second-line for sensitive relapsed SCLC
Satouchi *et al* (2014)	Phase Ⅲ trial: amrubicin+cisplatin (AP) *vs* irinotecan+cisplatin (IP) in chemotherapy-naive patients with extensive SCLC in Japan	AP is inferior to IP, being IP the standard treatment for extensive-stage SCLC
Sun *et al* (2016)	Phase Ⅲ trial: amrubicin+cisplatin (AP) *vs* etoposide and cisplatin (EP) for previously untreated SCLC in China.	AP therapy demonstrated non-inferiority to EP therapy, prolonging survival for 1.5 months
Trafalis *et al* (2016)	Phase Ⅱ trial: irinotecan+bevacizumab in relapsed chemo-resistant SCLC in Greece	Combination demonstrates promising efficacy and low toxicity compared to controls
Glisson *et al* (2017)	Phases Ib and Ⅱ multinational trials: rilotumumab or ganitumab or placebo+chemotherapy as first-line treatment in SCLC	Improved survival for rilotumumab. Rilotumumab or ganitumab + chemotherapy are tolerable, overall outcomes were not improved in patients with SCLC
SCLC: small cell lung cancer; *vs*: versus; AP: amrubicin+cisplatin; IP: irinotecan+cisplatin; EP: etoposide and cisplatin.						

**Table 2:** Phases Ⅱ or Ⅲ clinical trials related to the NSCLC treatment

Reference	Brief study methods	Relevant key findings
Paz-Ares *et al* (2018)	Phase Ⅲ multinational trial: pembrolizumab *vs* placebo. Both groups with carboplatin+paclitaxel in metastatic, squamous NSCLC	Addition of pembrolizumab to chemotherapy resulted in significantly longer overall survival and progression-free survival than chemotherapy alone
Weiss *et al* (2016)	Phase Ⅱ trial: pemetrexed, and bevacizumab for never or former/light smoking patients stage Ⅲb, Ⅳ non-squamous NSCLC in United States	Combination of the carboplatin, pemetrexed and bevacizumab demonstrated activity with acceptable toxicity
Ferry *et al* (2017)	Phase Ⅲ trial: platinum agent and dose of cisplatin in relation to chemo-naive stage Ⅲb/Ⅳ NSCLC patient outcomes in United Kingdom and Ireland	Gemcitabine+carboplatin is not inferior to cisplatin in terms of survival Carboplatin with more adverse events and cisplatin with worse survival
Palussiere *et al* (2018)	Phase Ⅱ trial: survival outcomes of percutaneous radiofrequency ablation (RFA) for patients with stage Ⅰa NSCLC, ineligible for surgery in France	RFA: efficient, well tolerated, does not adversely affect pulmonary function and survival is comparable to that of stereotactic body radiotherapy
Camerini *et al* (2015)	Phase Ⅱ trial: oral vinorelbine in chemotherapy naive elderly (≥70 years) PS 0-2 patients with stage Ⅲb to Ⅳ NSCLC in Italy	Safe in elderly patients with long-term disease stabilization coupled with an optimal patient compliance
Katsaounis *et al* (2015)	Phase Ⅱ trial: metronomic vinorelbine in combination with cisplatin as first-line treatment in inoperable stage Ⅲb or stage Ⅳ NSCLC in Greece	The combination is active, although myelotoxic, therapeutic option in the first-line setting
Ikeda *et al* (2018)	Phase Ⅱ trial: combination therapy of bevacizumab, cisplatin, and docetaxel, followed by bevacizumab as maintenance in chemotherapy-naive with stages Ⅲa, Ⅲb and Ⅳ NSCLC in Japan	The combination therapy was highly effective, despite the high incidence of grade 3/4 neutropenia
Socinski *et al* (2018)	Phase Ⅲ multinational trial: atezolizumab+bevacizumab+chemotherapy in metastatic non-squamous NSCLC without previously chemotherapy	The combination significantly improved progression-free survival and overall survival, regardless of PD-L1 expression
Hellmann *et al* (2018)	Phase Ⅲ multinational trial: nivolumab+ipilimumab *vs* chemotherapy in stage Ⅳ or recurrent NSCLC	Progression-free survival significantly longer for combination than chemotherapy, irrespective of PD-L1 expression level
Reck *et al* (2016)	Phase Ⅲ multinational trial: pembrolizumab *vs* platinum-based chemotherapy in untreated stage Ⅳ NSCLC, with PD-L1 expression on at least 50% of tumor cells	Pembrolizumab allowed significantly longer progression-free and overall survival and with fewer adverse events
Gandhi *et al* (2018)	Phase Ⅲ multinational trial: pemetrexed and a platinum-based drug plus either pembrolizumab or placebo in metastatic nonsquamous NSCLC without previous treatment for metastatic disease	Pembrolizumab+standard chemotherapy resulted in significantly longer overall survival and progression-free survival than chemotherapy alone
Sandler *et al* (2000)	Phase Ⅲ trial: gemcitabine+cisplatin *vs* cisplatin alone in chemotherapy-naive patients with unresectable stage Ⅲa, Ⅲb, or Ⅳ NSCLC in United States.	Gemcitabine+cisplatin is superior in terms of response rate, time to disease progression, and overall survival
Park *et al* (2007)	Phase Ⅲ trial: additional four or two more cycles of third-generation, platinum-doublet treatment for stages Ⅲb to Ⅳ NSCLC resistant to chemotherapy in South Korea	Similar overall survival with four or six cycles of chemotherapy with favourable time to progression for six cicles
Pujol *et al* (2014)	Phase Ⅲ multinational trial: pemetrexed maintenance *vs* placebo in advanced non-squamous NSCLC	Low incidence of low-grade toxicities with long-term pemetrexed exposure without compromising quality of life
Paz-Ares *et al* (2013)	Phase Ⅲ multinational trial: pemetrexed continuation maintenance *vs* placebo in advanced non-squamous NSCLC	Pemetrexed is well-tolerated and offers superior survival, also an efficacious treatment for patients who did not progress during pemetrexed-cisplatin induction therapy
Lee *et al* (2015)	Phase Ⅱ multinational trial: pemetrexed+dexamethasone, folic acid, and vitamin B12+erlotinib *vs* erlotinib *vs* pemetrexed in EA and non-EA never-smoker patients and patients with advanced or metastatic non-squamous NSCLC	Better progression-free survival for pemetrexed-erlotinib in EA patients
van Kruijsdijk *et al* (2016)	Phase Ⅱ multinational trial: pemetrexed+carboplatin *vs* single-agent pemetrexed in the second-line treatment of stages Ⅲb and Ⅳ NSCLC	Combination benefited most women, stage Ⅳ, high body mass index and/or adenocarcinoma. Individualized treatment can improve clinical outcome
Ellis *et al* (2015)	Phase Ⅱ multinational trial: volasertib monotherapy or+pemetrexed *vs* pemetrexed monotherapy in recurrent, advanced, or metastatic NSCLC after previous platinum-based chemotherapy	The combination did not increase toxicity but also did not improve efficacy compared with single-agent pemetrexed
Paz-Ares *et al* (2017)	Phase Ⅲ multinational trial: ramucirumab+docetaxel *vs* docetaxel alone in squamous or non-squamous stage Ⅳ NSCLC	Favourable overall survival and manageable safety for combination
Reck *et al* (2017)	Phase Ⅲ multinational trial: docetaxel+ramucirumab *vs* placebo in refractory patients stage Ⅳ NSCLC	Combination is an appropriate treatment option even in this difficult-to-treat population
Rittmeyer *et al* (2017)	Phase Ⅲ multinational trial: atezolizumab *vs* docetaxel in previously treated squamous or non-squamous NSCLC	Atezolizumab treatment resulted in a relevant improvement of overall survival, regardless of PD-L1 expression or histology, with a favourable safety profile
Borghaei *et al* (2015)	Phase Ⅲ multinational trial: nivolumab *vs* docetaxel in previously treated squamous or non-squamous NSCLC	Overall survival longer with nivolumab than with docetaxel for advanced previously treated non-squamous NSCLC
Herbst *et al* (2016)	Phase Ⅱ/Ⅲ multinational trial: pembrolizumab *vs* docetaxel in previously treated PD-L1-positive, advanced NSCLC	Pembrolizumab prolongs overall survival and has a favourable benefit-to-risk profile in previously treated patients
Neal *et al* (2016)	Phase Ⅱ trial: erlotinib, cabozantinib, or erlotinib and cabozantinib in advanced non-squamous NSCLC in United States	Cabozantinib alone or+erlotinib has clinically meaningful, superior efficacy over erlotinib alone, with additional generally manageable toxicity
NSCLC: non-small cell lung cancer; vs: versus; RFA: radiofrequency ablation; PS: performance status; PD-L1: programmed death-ligand 1; EA: East Asian.		

**Table 3:** Phases Ⅱ or Ⅲ clinical trials with focus on advanced NSCLC when molecular profile for gene mutations are positive

Reference	Brief study methods	Relevant key findings
Paz-Ares *et al* (2017)	Phase Ⅱb multinational trial: afatinib *vs* gefitinib in treatment-naive patients with stage Ⅲb/Ⅳ NSCLC and a common *EGFR* mutation	Progression-free survival, time-to-treatment failure and objective response rate were significantly improved with afatinib with no significant difference in overall survival
Soria *et al* (2018)	Phase Ⅲ multinational trial: osimertinib *vs* gefitinib or erlotinib in previously untreated *EGFR* mutation-positive in advanced or metastatic NSCLC	Osimertinib showed superior efficacy with a similar safety profile and lower rates of serious adverse events
Wu *et al* (2017)	Phase Ⅲ multinational trial: oral dacomitinib *vs* oral gefitinib in *EGFR*-mutation-positive newly diagnosed advanced NSCLC	Dacomitinib significantly improved progression-free survival over gefitinib in first-line treatment
Reungwetwattana *et al* (2018)	Phase Ⅲ multinational trial: osimertinib *vs* standard EGFR tyrosine kinase inhibitors in locally advanced or metastatic *EGFR*-mutated NSCLC	Osimertinib has CNS efficacy and reduced risk in patients with untreated *EGFR*-mutated NSCLC
Seto *et al* (2014)	Phase Ⅱ trial: erlotinib+bevacizumab *vs* erlotinib alone in stage Ⅲb/Ⅳ or recurrent non-squamous NSCLC with activating *EGFR* mutation-positive disease in Japan	Combination presented better median progression-free survival and serious adverse events occurred at a similar frequency in both groups
Barata *et al* (2016)	Phase Ⅱ trial: erlotinib in metastatic NSCLC with activating mutations in the tyrosine kinase (TKI) domain of the EGFR in Portugal	Similar results compared with other clinical trials in Caucasian patients
Gridelli *et al* (2016)	Phase Ⅲ trial: erlotinib+bevacizumab *vs* erlotinib in advanced NSCLC harboring activating *EGFR* mutations in Italy	The combination seems to be the best first-line treatment
Janne *et al* (2014)	Phase Ⅱ multinational trial: dacomitinib as initial systemic therapy in stage Ⅲb/Ⅳ NSCLC adenocarcinoma *EGFR*-mutant	Only 6% of patients discontinued dacomitinib due to adverse event. Dacomitinib was associated with clinically meaningful improvements in multiple disease-related symptoms early on, and these improvements were maintained over time
Yoshimura *et al* ^[82]^ (2015)	Phase Ⅱ trial: gefitinib and pemetrexed as first-line chemotherapy in *EGFR*-mutated NSCLC in Japan	Combination showed a high overall response rate, long median progression-free survival and acceptable toxicity
Shaw *et al* (2013)	Phase Ⅲ trial: crizotinib *vs* intravenous pemetrexed or docetaxel in locally advanced or metastatic ALK-positive lung cancer in United States	Crizotinib is superior including progression-free survival, response rate, symptoms of lung cancer and global quality of life
Solomon *et al* (2018)	Phase Ⅲ multinational trial: crizotinib *vs* pemetrexed+cisplatin or carboplatin as first-line treatment in advanced ALK-positive non-squamous NSCLC	Crizotinib allowed longest overall survival
Soria *et al* (2017)	Phase Ⅲ multinational trial: ceritinib *vs* platinum-based chemotherapy in stage Ⅲb/Ⅳ ALK rearranged non-squamous NSCLC	Ceritinib showed a marked improvement in progression-free survival
Novello *et al* (2018)	Phase Ⅲ multinational trial: alectinib *vs* platinum-based chemotherapy in advanced/metastatic ALK-positive NSCLC patients previously treated with platinum-based doublet chemotherapy and crizotinib	Alectinib significantly improved systemic and CNS efficacy and grade ≥3 adverse events were more common with chemotherapy
Planchard *et al* (2017)	Phase Ⅱ multinational trial: dabrafenib+trametinib in *BRAF*(V600E)-mutant metastatic NSCLC	Combination presented a clinically meaningful antitumour activity and a manageable safety profile
Hyman *et al* (2015)	Phase Ⅱ multinational trial: vemurafenib in *BRAF* V600 mutation-positive nonmelanoma cancers including NSCLC	Vemurafenib presented modest antitumor activity
Soria *et al* (2017)	Phase Ⅱ multinational trial: docetaxel+selumetinib *vs* placebo in *KRAS*-mutant advanced NSCLC	Combination showed no clinical benefit compared with docetaxel alone
Hirano *et al* (2017)	Phase Ⅱ trial: erlotinib low dose as maintenance treatment after platinum doublet chemotherapy in NSCLC harboring *EGFR* mutation in Japan	Study was stopped early due to poor accrual with the suggestion that maintenance therapy with low-dose erlotinib might be useful and tolerable
Paz-Ares *et al* (2015)	Phase Ⅲ multinational trial: orafenib or matching placebo in advanced relapsed/refractory, wild-type or mutated *KRAS* NSCLC	Third-/fourth-line sorafenib therapy increased progression-free survival but not overall survival
ALK: anaplasic lymphoma kinase; EGFR: epidermal growth factor receptor; KRAS: Kirsten rat sarcoma; CNS: central nervous system.		

## Discussion

**Figure 2:**
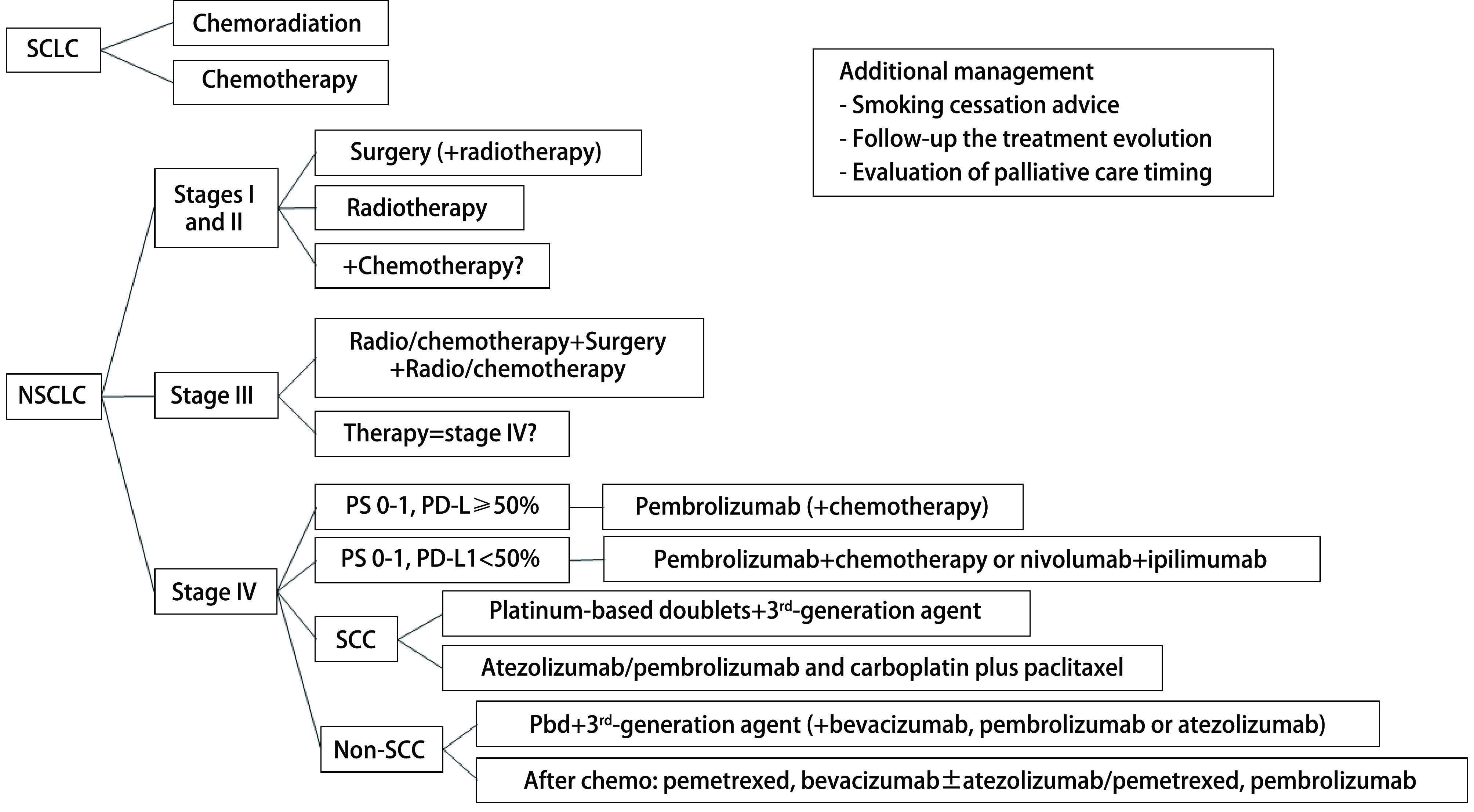
Brief summary of lung cancer treatment options.

Lung cancer can be divided in two major histological types: SCLC^[23]^ and NSCLC^[23]^. The NSCLC accounts more than 80% of all lung cancer and it comprises 2 major types: nonsquamous (e.g.: adenocarcinoma, large-cell carcinoma, and other cell types); and squamous cell carcinoma, being dived in stages 0 to Ⅳ ^[4, 24, 25]^. Some of the lung cancer main treatment options, according to the literature, are depicted in Fig 2.

Pbd: platinum-based, doublets; Chemo: chemotherapy.

The SCLC is a very chemosensitive tumor and therapeutics is usually based on combined chemoradiation for tumors confined to the chest and palliative chemotherapy for advanced or metastatic disease. Surgery is generally not recommended in the SCLC management due to the high risk of recurrence. For extensive SCLC, atezolizumab combined with cisplatin and etoposide is the only association that can improve the overall survival, although it is not approved by regulatory agencies worldwide^[26]^. Cisplatin plus irinotecan can be used in the subsequent treatment for patients with sensitive relapsed SCLC, because of better efficacy and longer overall survival than the single-agent topotecan. The association of amrubicin to cisplatin is a promising treatment option for Chinese patients. Alternatively, pembrolizumab or nivolumab plus ipilimumab can be employed in patients with a high tumor mutational burden, not previously treated with immunotherapy^[14, 27-31]^. Other promising targeted therapeutics includes talazoparib, veliparib and rovalpituzumab tesirine. Treatment through the combination of rilotumumab and ganitumab with platinum-based chemotherapy is also being studied for those patients with extensive stage SCLC^[32]^. Selected studies of phases Ⅱ or Ⅲ clinical trials are summarized in Tab 1.

Except for stage 0, that is considered "*in situ*" and completely surgically removed, the NSCLC treatment is much more complex and will be described according to its major stages (Ⅰ to Ⅳ) classification.

### Treatment algorithm for stage Ⅰ NSCLC

Stage Ⅰ NSCLC treatment is usually more invasive than stage 0. The treatment includes respiratory affected tissue removal through surgery together with compromised lymph nodes and pleura. Surgery, whenever possible, is still the best choice to manage stage Ⅰ NSCLC. The extension of the tumor will influence in the surgical technique:

- For healthy patients, stage Ⅰb, lobectomy or anatomic pulmonary resection together with mediastinal lymph node dissection is the preferential treatment.

- Surgical treatment should be less aggressive through sublobar resection when the lesion is inferior 1 cm and presents mostly ground glass opacity, or in those patients with comorbidities and decreased pulmonary function.

- If, after first surgery, there are still positive margins a new resection followed by radiotherapy, whenever possible, should be performed^[15, 33-36]^.

For severe illness patients, medically inoperable, the radiotherapy such as stereotactic body radiation therapy (SBRT) or radiofrequency ablation (RFA) may be the first treatment option. However, when tumor is completely resected, postoperative radiotherapy is not routinely recommended^[15, 34, 37-39]^. Chemotherapy can be used in the preoperative period with positive results since it can reduce the tumor size. Furthermore, the overall survival, time to distant recurrence, and recurrence free can be significantly improved^[23]^.

### Treatment algorithm for stage Ⅱ NSCLC

Stage Ⅱ NSCLC patients are treated in the same basis of the stage Ⅰ, but again, more invasively if the health of the patient allows respiratory resection surgery including lymph nodes. Surgery, whenever possible, is still the best choice to manage stage Ⅱ. The extension of the tumor will influence in the surgical technique:

- For healthy patients, stage Ⅱ, lobectomy or anatomic pulmonary resection together with mediastinal lymph node dissection is recommended^[15, 34]^.

- If, after first surgery, there are still positive margins, a new resection followed by radiotherapy, whenever possible, should be performed. The adjuvant treatment with four cycles of cisplatin-based chemotherapy can increase the overall survival for completely resected tumors^[15, 33, 34]^. The reduction of the cisplatin can improve the quality of life, however it is not recommended because of the worsening in survival^[38]^. Higher risk patients should be treated as escribed in stage Ⅰ.

Due to the limited benefits, chemotherapy and radiotherapy are generally not recommended. For severe illness patients, with node negative tumors ≤5 cm and those older than 75 years, the stereotactic ablative radiotherapy may be an option. This treatment choice should be discussed with patients since it can decrease survival^[34, 40, 41]^.

### Treatment algorithm for stages Ⅲa Ⅲb and Ⅲc NSCLC

Since there are no specific guidelines to determine to what extent lung tumors should be considered resectable or unresectable disease, an experienced multidisciplinary team is required in order to plan the treatment sequence for the heterogeneous and complex stage Ⅲ NSCLC. Patients should undergo to an accurate imaging diagnostic and receive brain imaging for initial staging. For presumably resectable stage Ⅲa, induction therapy (radiation/chemotherapy) followed by surgery, according to the extension of the tumor and the patient´s health, might be better than surgery alone. If the tumor is surgically removed the following therapy will probably include 4 cycles of adjuvant cisplatin-based chemotherapy with subsequently radiation to improve overall survival^[15, 34, 42-44]^.

If, after first surgery, there are still positive margins a new resection followed by radiotherapy, whenever possible, should be performed. The adjuvant treatment with cisplatin-based chemotherapy can increase the overall survival for completely resected tumors^[15, 33, 34]^.

Stages Ⅲb, Ⅲc and some Ⅲa (multiple nodal involvement) are usually unresectables, being not possible to completely remove the tumors only by surgery. The more invasive procedure will also be conditioned by the health status. For medically fit patients the concurrent chemoradiotherapy with cisplatin-based chemotherapy, usually with etoposide or vinorelbine, is the first choice. Metronomic oral vinorelbine, although myelotoxic, promotes a safe long-term disease stabilization, being well-tolerated in elderly patients. The recommended radiotherapy is 60 Gy-66 Gy in 30-33 fractions over 6-7 weeks. When concurrent treatment is not possible, sequential chemotherapy followed by definitive radiotherapy is indicated. Durvalumab is an option for stage Ⅲ NSCLC with PD-L1 expression equal or superior to 1%, after achieving disease control with platinum-based chemoradiation^[15, 34, 45, 46]^. When patients are unsuitable for curative radiotherapy, the therapy should be based on stage Ⅳ treatment as described in the next section^[47]^.

### Treatment algorithm for stage Ⅳ NSCLC

The widespread metastasis turns the stage Ⅳ NSCLC very difficult to be managed. The first treatment choice will take many aspects in consideration that must be discussed in a multidisciplinary team, in order to choose the best individualized option. In general, systemic therapy (including targeted therapy and immunotherapy), clinical trials, and/or palliative care will be the treatment choice, according to the extension of the disease and the patient health status^[4]^.

Tumor mutational burden is a promise biomarker for immune checkpoint blockade efficacy, mainly in patients with PD-L1 negative. The immunotherapy treatment is more responsive when PD-L1 tumor levels are high^[48]^. When PD-L1 expression is ≥50% pembrolizumab can be a first option as monotherapy. Pembrolizumab plus chemotherapy is the standard of care, irrespective of PD-L1 expression. Bevacizumab plus chemotherapy was the standard of care before immunotherapy, although it is contraindicated for squamous-cell tumors, bleeding high risk patients, or when the tumor is near large blood vessels. Bevacizumab plus chemotherapy combined with atezolizumab also improves outcomes as first-line treatment for nonsquamous metastatic NSCLC patients^[3, 4, 9, 49]^. Nivolumab plus ipilimumab can improve outcomes and should be considered for first-line treatment^[50]^.

Excision repair cross-complementation group 1 (ERCC1) low expression from Ⅲb to Ⅳ NSCLC is related to favorable treatment with cisplatin-based chemotherapy. Furthermore, ERCC1-positive tumors presents benefits in progression-free survival when treated with erlotinib and bevacizumab^[51]^.

Treatment algorithms for stage Ⅳ NSCLC when molecular tests for gene mutations are negative:

If PS 0-1 and PD-L1≥50% of tumor cells: pembrolizumab monotherapy is the first treatment option, irrespective of histology, since this drug presents better overall survival with fewer adverse events and lower risk of death than platinum-based chemotherapy^[4, 8, 9, 18, 52-55]^. Combination of immunotherapy plus platinum-based chemotherapy may be considered due its increase in response rate^[3, 9, 16, 49, 56]^.

If PS 0-1 and PD-L1 < 50% or unknown: the standard of care is pembrolizumab plus patinum-based chemotherapy regardless of tumor histology, followed by pembrolizumab maintenance therapy (pembrolizumab plus pemetrexed for non-squamous tumors)^[8, 34, 38, 57]^. Alternatively, and irrespective of PD-L1 expression, nivolumab associated with ipilimumab can be used in patients who do not tolerate chemotherapy or wish to preserve chemotherapy as a future treatment option^[8, 14, 58]^. Atezolizumab plus bevacizumab combined with platinum-based chemotherapy is also an acceptable option^[49]^.

Squamous cell carcinoma (SCC): Four cycles of platinum-based doublets (up to 6 cycles in selected cases) with the addition of a third-generation cytotoxic agent (gemcitabine, vinorelbine, taxanes) therapeutics is recommended^[34, 59, 60]^. Atezolizumab or pembrolizumab and carboplatin plus paclitaxel or nab-placlitaxel/carboplatin (better overall response rate and tolerability than sb-paclitaxel/carboplatin) presents better results than only chemotherapy, regardless of PD-L1 expression^[9, 34, 49, 61]^.

Non-squamous-cell carcinoma (non-SCC): Platinum-based doublet with a third-generation agent is recommended. The addition of bevacizumab, pembrolizumab or atezolizumab in the treatment of selected patients can increase the overall survival^[9, 34, 49, 56, 60, 62]^. After chemotherapy, pemetrexed and bevacizumab±atezolizumab or pemetrexed and pembrolizumab can be used as long-term in the maintenance of stable disease, with no important safety concerns, being well-tolerated and increasing overall survival for patients with good performance status, after no progression with pemetrexed-cisplatin^[63-65]^. Nivolumab plus ipilimumab can improve outcomes compared to chemotherapy for high tumor mutation burden patients, although it was not approved by regulatory agencies worldwide^[14]^. Selected studies of phases Ⅱ or Ⅲ clinical trials are summarized in Tab 2.

Treatment algorithms for stage Ⅳ NSCC when molecular tests for gene mutations are positive:

The treatment standard of care should include tumor molecular profiling. The most predictive biomarkers are *ALK*, *ROS1* gene rearrangements, sensitizing *EGFR* mutations, *HER2* and *BRAF* V600E, Kirsten rat sarcoma (*KRAS*) mutations, *RET* gene rearrangements, and high-level *MET* amplifications^[4, 66, 67]^. For these genetic alterations, molecular profiling with targeted therapies are considered the first treatment choice. However, there are no personalized targeted therapy approved for some of these mutations and the first treatment choice is still chemotherapy^[66, 68]^.

EGFR pathway is present in most of NSCLC and leads to the continuous increase of the tumor through angiogenesis, invasion, metastasis and inhibition of apoptosis. Thus, when the mutation is positive, the therapeutics may intent to block the EGFR^[1, 69]^. For mutations in the *EGFR* discovered prior to first-line chemotherapy, the treatment can be performed by using erlotinib, gefitinib, afatinib, osimertinib or dacomitinib. If the mutation is discovered during first-line chemotherapy, this initial treatment and maintenance therapy should be finished. Alternatively, chemotherapy can be substituted by erlotinib, afatinib or gefitinib. Furthermore, when compared to chemotherapy, this therapeutic allow a better quality of life. When comparing these drugs, osimertinib and dacomitinib has shown better overall survival with less toxicity. The overall survival can also be slight improved by the combinations of bevacizumab and erlotinib or of pemetrexed-carboplatin and gefitinib. In addition, osimertinib has a good progression-free survival among patients with central nervous system (CNS) metastasis^[4, 14, 34, 70-82]^.

If the positive gene is the *ALK* or *ROS1*, the first treatment inhibitors can be crizotinib (unique option for patients with *ROS1* mutation), ceritinib, alectinib or brigatinib, presenting better results than chemotherapy. Crizotinib presents few side effects and a very high response in patients with positive ALK advanced NSCLC, including those with brain metastases. However, due to possible adverse effects, close monitoring of liver function is recommended when using crizotinib. First-line alectinib improved outcomes compared to first-line crizotinib. Alternatively, if these drugs are not tolerated or ineffective, brigatinib or lorlatinib can be used in trials, since they are not approved by regulatory agencies worldwide^[1, 4, 14, 16, 34, 83-89]^.

When the changes affect the *BRAF* gene (V600E), the treatment can be the combination of dabrafenib and trametinib. If BRAF/MEK inhibitor where used in first-line treatment, platinum-based chemotherapy can be used in the subsequent therapy^[14, 16, 90, 91]^.

The most common lung cancer oncogenic alteration mutation is in the *KRAS*, being related to smoking and poor prognosis in NSCLC. There is not any targeted-therapy for *KRAS*-mutated patients^[4, 66, 68, 92]^. Selected studies of phases Ⅱ or Ⅲ clinical trials are summarized in Tab 3.

### Additional management

Smoking cessation must be advised in any stage of the disease since it can improve the outcomes of the treatment because of the interaction with the employed drugs. The preferred approach includes behavior techniques along with pharmacotherapy. Furthermore, stop smoking improves quality of life by reducing the "guilty" feeling. A follow-up is also advised to close observe the evolution of the treatment, as well as, to identify complications, health and mental status. It is also of paramount importance to evaluate the palliative care timing, mainly for patients with advanced disease^[14-16, 34]^.

### Subsequent therapy

When lung cancer does not stop developing during therapeutics, or recurs after first treatment, the subsequent management will be based on tumor and patient characteristics, as well as, modalities of previous approaches. In subsequent therapy, all molecular tests not performed before are recommended. If lung cancer continues to develop during chemotherapy, as the first treatment, subsequent therapy most often consists of a single drug such as pemetrexed or docetaxel^[4, 34, 93, 94]^. However, the association of docetaxel with nintedanib or ramucirumab presents better efficacy with manageable toxicity. On the other hand, the association of docetaxel plus a targeted drug such as selumetinib presents no benefits and should be avoided. Ramucirumab presents contra-indications due to the high risk of uncontrolled hypertension with severe hemorrhage, gastrointestinal perforation, bleeding or fistula. Thus, potential risks and benefits must be weighted before choosing this modality of treatment^[4, 95-98]^. The treatment with immunotherapeutic agents are justified in subsequent therapy because of the improvement in the overall survival, longer duration of response and less toxicity when compared with cytotoxic chemotherapy^[16, 34, 93]^.

For metastatic non-SCC and SCC with no prior immunotherapy, single-agent pembrolizumab is a good option, with manageable side effects and prolonged overall survival in PD-L1-positive previously treated patients. Nivolumab or atezolizumab are recommended regardless of PD-L1 expression in order to improve overall survival with a favorable safety profile over docetaxel^[4, 16, 34, 49, 99-102]^. In addition, anti-PD-1/PD-L1 antibodies treatment presents less toxicity (most common events being hypothyroidism, hyperthyroidism, skin rash, pneumonitis, and hepatitis) and better overall survival, progression free survival and overall response rate than docetaxel, mainly for higher levels of PD-L1 expression, and even when PD-L1 expression is < 1%^[4, 22, 103]^.

Additionally, osimertinib is recommended in patients with metastatic EGFR T790M-positive NSCLC that has progressed on erlotinib, gefitinib, or afatinib therapy^[4, 104-106]^. The combination of cabozantinib plus erlotinib for second or third-line treatments presents better efficacy, with manageable additional toxicity, than monotherapy with erlotinib for EGFR wild-type NSCLC patients^[107]^. Monotherapy with sorafenib, despite increasing progression-free survival did no improve overall survival when used as a third-/fourth-line therapy^[108]^. Finally, new predictive biomarkers are expected to be developed in order to improve treatment individualization allowing the greatest benefit^[54, 68, 109, 110]^.

### Clinical points

In summary, SCLC therapeutics is usually based on chemoradiation, immunotherapy palliative chemotherapy for advanced or metastatic disease and surgery is generally not recommended. Extensive SCLC can be managed with immunotherapy associated or not with chemotherapy.

Except for stage 0, that is considered "*in situ*" and completely surgically removed, the NSCLC treatment is complex. Stage Ⅰ NSCLC treatment is usually surgical and the extension of the tumor will influence in the surgical technique and the complementary radiotherapy. Preoperative chemotherapy has potential to reduce the tumor size. Stage Ⅱ patients are treated more invasively in the same basis of the stage Ⅰ. For stage Ⅲ, if the tumor is surgically removed the following therapy will probably include chemotherapy with subsequently radiotherapy. When unresectable, chemoradiation with chemotherapy is the first choice. Immunotherapy associated or after chemotherapy can be an option. Stage Ⅳ represents a challenge and in general, systemic therapy, clinical trials, and/or palliative care will be the treatment choice, according to the histology, molecular tests for gene mutation, extension of the disease and the patient health status.

## Conclusions

Up to now, despite the improvement in the overall survival, longer duration of response and toxicity reduction, there are still many gaps in the NSCLC treatment strategy algorithm, including the drug´s optimal doses and the optimal sequencing of immunotherapy and chemotherapy, when use associations, the role of vaccines, ideal duration of treatment, most appropriate approach to elderly and patients with poor performance status, and patients that eventually acquire resistance even after a personalized therapy. In addition, due to the burden of increasing costs, the benefits of some associations of target therapies and immunotherapy are questionable. In this context, new predictive biomarkers are expected to be developed in order to improve treatment individualization allowing the greatest benefit.

## Author contributions

De Mello RA and Pozza DH designed the study and were responsible for articles selection, respective data collection and evaluation. Pozza DH wrote the manuscript draft and performed the statistical analysis. De Mello RA supervised the research, provided suggestions for the improvement of the study and finalized the manuscript. All the authors had access to the data. All authors read and approved the final manuscript as submitted.
